# Glycated albumin and continuous glucose monitoring metrics across pregnancy in women with pre‐gestational diabetes

**DOI:** 10.1002/edm2.376

**Published:** 2022-09-19

**Authors:** Johanne Holm Toft, Ingvild Dalen, Øyvind Skadberg, Lasse Gunnar Gøransson, Inger Økland, Inger Hjørdis Bleskestad

**Affiliations:** ^1^ Department of Obstetrics and Gynecology Stavanger University Hospital Stavanger Norway; ^2^ Department of Clinical Science University of Bergen Bergen Norway; ^3^ Section of Biostatistics, Department of Research Stavanger University Hospital Stavanger Norway; ^4^ Department of Medical Biochemistry Stavanger University Hospital Stavanger Norway; ^5^ Department of Internal Medicine Stavanger University Hospital Stavanger Norway; ^6^ Department of Clinical Medicine University of Bergen Bergen Norway; ^7^ Department of Caring and Ethics University of Stavanger Stavanger Norway

**Keywords:** continuous glucose monitoring, glycated albumin, glycated haemoglobin A1c, pregnancy, type 1 diabetes, type 2 diabetes

## Abstract

**Introduction:**

Glycated albumin (GA), a biomarker reflecting short‐term glycaemia, may be useful to assess glycaemic control in pregnancy. We examined the association between GA and continuous glucose monitoring (CGM) metrics across gestation.

**Methods:**

In this prospective cohort study including 40 women with pre‐gestational diabetes, blood samples for analysis of GA and glycated haemoglobin A1c (HbA1c) were collected at pregnancy week 12, 20, 24, 28, 32 and 36. In the CGM‐group (*n* = 19), CGM data were collected from first trimester until pregnancy week 36. Receiver operating characteristic (ROC) curves were used to assess the accuracy of GA and HbA1c to detect poor glycaemic control, using CGM metrics as the reference standard. This study was conducted at Stavanger University Hospital, Norway, in 2016–2018.

**Results:**

Glycaemic control improved across gestation with more time spent in target range, coinciding with decreased glycaemic variability and lower mean GA level. There was statistically significant correlation between GA and most CGM metrics. The area under the ROC curves (AUC) for detecting time in range <70% and time above range >25% for the pregnancy glucose target 63–140 mg/dl (3.5–7.8 mmol/L) were 0.78 and 0.82 for GA, whereas AUCs of 0.60 and 0.72 were found for HbA1c, respectively.

**Conclusions:**

Higher GA levels were associated with less time spent in target range, more time spent in the above range area and increased glycaemic variability. GA was more accurate than HbA1c to detect time above range >25% and time in range <70%.

## Graphical abstract

In our prospective cohort study in pregnant women with diabetes, glycated albumin correlated well with CGM metrix. Glycated albumin was more accurate than HbA1c to detect time above range >25% and time in range <70%.

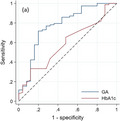

## 1 INTRODUCTION

In women with pre‐gestational diabetes, the risk of adverse pregnancy outcomes correlate with the level of glycaemia.^1^ In Norway, the prevalence of pregnancies complicated by pre‐gestational diabetes has been stable around 0.7% for the last decade.^2^ Corresponding numbers are reported in Australia (0.6%) and in the United States (0.9%).^3^, ^4^ However, due to increasing obesity, earlier onset of type 2 diabetes (T2D) and higher maternal age, the prevalence of pre‐gestational diabetes is expected to rice globally.

Continuous glucose monitoring (CGM) enables users to monitor their glucose level, providing the opportunity to respond to glucose fluctuations as they occur.^5^ With randomized controlled trials showing that CGM is associated with improvements in maternal glycaemic control and neonatal outcomes,^6^ the use of CGM in antenatal care is increasing.^7^ By recent international consensus for CGM monitoring, the pregnancy glucose target range for type 1 diabetes (T1D) was set to 63–140 mg/dl (3.5–7.8 mmol/L). Women should strive to achieve >70% of time within target range.^8^ Currently, there are not provided CGM targets for pregnant women with T2D, due to the lack of evidence and limited data. However, access to CGM for all pregnant women with diabetes is still limited.

Glycated albumin (GA), a biomarker reflecting short‐term glycaemia (2–4 weeks) has been suggested to supplement glycated haemoglobin A1c (HbA1c) in monitoring glycaemic control.^9^ In diabetic pregnancies where strict glycaemic control is important to reduce adverse maternal/foetal outcomes, a marker reflecting recent glycaemic status is preferable. Moreover, GA may be better than HbA1c to detect glucose variability and fluctuations, which have been associated with increased risk of developing large for gestational age (LGA) foetuses.^10^ Furthermore, elevated maternal GA levels may predict perinatal complications.^11^ Thus, GA may be a useful tool for detecting and monitoring recent glycaemic control in diabetic pregnancies, and in particular, the glucose fluctuations, not provided by HbA1c.

Haemoglobin A1c is recognized as the gold standard of diabetic survey^12^ and was included as a diagnostic criterion for diabetes mellitus in 2011.^13^ HbA1c reflects mean glycaemia over the preceding 8–12 weeks.^14^ There is a linear relationship between average glucose and HbA1c in pregnancy, but the change in HbA1c reflects a smaller difference in mean glucose compared with that found in non‐pregnant adults.^15^ Moreover, altered erythrocyte turnover and iron deficiency may influence HbA1c, making it less accurate during pregnancy.^16^, ^17^ Despite these limitations, HbA1c is used worldwide in clinical practice to monitor glycaemic control during pregnancy.

Recently, a new high‐throughput method for GA measurement using liquid chromatography–tandem mass spectrometry (LC–MS/MS) was developed in our laboratory.^18^ Subsequently, the reference interval for GA in healthy pregnant women was established.^19^

The primary aim of this study was to explore the association between GA and CGM metrics across gestation in women with pre‐gestational diabetes. Secondly, we investigated the accuracy of GA and HbA1c to detect poor glycaemic control using CGM metrics as the reference standard.

## 2 METHODS

### 2.1 Study population

This prospective cohort study was conducted at Stavanger University Hospital, Norway, in 2016–2018. Women were asked to participate in the study when they met at the antenatal diabetic outpatient clinic in first trimester. All women with pre‐gestational diabetes and singleton pregnancies were eligible for inclusion. In Norway, antenatal care of women with pre‐existing diabetes is primarily organized in specialist health care where the woman meets an obstetrician, a midwife and an endocrinologist at every visit. All participants received current routine clinical care, with antenatal visits every 2–4 weeks until pregnancy week 38. Women with otherwise uncomplicated pregnancies, had an additional consultation at pregnancy week 39 and labour was induced no later than the due date. In addition, the consenting women had blood samples for analysis of GA and HbA1c taken at Stavanger University Hospital's Clinical Trial Ward around pregnancy week 12, 20, 24, 28, 32 and 36, coordinated with the clinical appointments.

Blood samples for GA were collected in serum gel tubes, stored at room temperature for 30 min, centrifuged at 2500 *g* to obtain serum, and stored at −75°C until used. GA was analysed by LC–MS/MS as previously described.^18^ HbA1c was analysed on BioRad Variant II Turbo, high‐performance liquid chromatography, standardized to the International Federation of Clinical Chemistry reference method (analytical variation ≤3%). All analyses were performed at the Department for Medical Biochemistry, Stavanger University Hospital.

### 2.2 Blood glucose data

According to recommendations in the Norwegian guideline, the HbA1c level should be <53 mmol/mol (<7%) in the preconception period and <42 mmol/mol (<6%) from second trimester. Throughout pregnancy, treatment goals for glucose are fasting plasma glucose 63–99 mg/dl (3.5–5.5 mmol/L) and <128 mg/dl (<7.1 mmol/L) 2 h postprandial.^20^ CGM were offered to women with poor glycaemic control, or additional challenges such as impaired awareness of hypoglycaemia. Otherwise, self‐monitoring of blood glucose with frequent daily measurements (7–10 times a day) was advised. In Norway, the use of CGM during pregnancy has markedly increased over the past years. Seventeen women in the study were already users of CGM before pregnancy, whereas four participants were offered CGM during pregnancy.

### 2.3 CGM system

Among the CGM users, the majority had Dexcom G4 (Dexcom Inc), whereas one had Freestyle Libre (Abbott) and another used the Medtronic CGM system (Medtronic). The Dexcom G4 device, measures subcutaneous interstitial glucose concentration every 10 s and generates a glucose value every 5 min, available for the user real time. Dexcom G4 requires calibration by the user against capillary plasma glucose twice daily. With the Freestyle Libre system, known as a ‘flash’ glucose monitor, no calibration is required. The interstitial glucose level is measured every 60 s, a glucose value is generated every 15 min, but the results are available only retrospectively when the sensor is scanned with a reading device. The Medtronic CGM system is also a real time system, generating a glucose value every 5 min.

### 2.4 Glucose data management

At every visit, available data from self‐monitored blood glucose and/or CGM were downloaded from the internet‐based Diasend system (Glooko). For the user of Medtronic CGM system, glucose data were downloaded from CareLink (Medtronic). We included CGM data from the 14 days leading up to each blood sampling at pregnancy week 12, 20, 24, 28, 32 and 36. According to recent consensus on CGM use, we required at least 70% coverage (percentage of time CGM is active) for inclusion in the analysis.^8^

From CGM data, we calculated mean glucose level and the percentage of time spent in target range (time in range, TIR), time below range (TBR) and time above range (TAR) for the pregnancy glucose target range 63–140 mg/dl (3.5–7.8 mmol/L).^8^ We also calculated time below range <54 mg/dl (<3.0 mmol/L), denoted TBR2. Measures of glycaemic variability included glucose standard deviation (SD) and coefficient of variation (CV).^8^

### 2.5 Obstetric data and outcomes

Information concerning pregnancy outcome was collected from medical records after delivery. Frequencies of small for gestational age and large for gestational age were calculated using the 10th and 90th percentile according to Gjessing et al.^21^ In addition, birth weight centiles and percentage birth weight deviations from the median birth weight for gestational age, were calculated.^21^

### 2.6 Ethical considerations/approval

The study was carried out in accordance with the Helsinki Declaration and was approved by the Regional Committees for Medical and Health Research Ethics, Western Norway (May 2016, REK 2016/563). The study was registered in Clinical Trials with identifier NCT 03330951. All included women received written information about the study and gave informed consent.

### 2.7 Statistical analyses

Categorical data are shown as percentages. Continuous variables are presented as mean with SD, or median with interquartile ranges (IQR) for skewed distributions. Differences in clinical characteristics between the CGM and non‐CGM group were assessed using independent samples *t*‐test (normal distribution) and Mann–Whitney test (skewed distribution) for continuous data, whereas Chi‐squared test was performed for categorical data. A *p*‐value < .05 was considered statistically significant.

Mean values of GA and HbA1c at different time points were estimated in mixed linear models with random intercepts and random effects of time points. Comparison of levels between time points was performed with paired samples *t*‐tests.

Correlation coefficients were used to assess relationships between GA, HbA1c and CGM metrics. The correlation coefficients were estimated allowing for the repeated measures design using the approach outlined by Hamlett et al.^22^ Confidence intervals (CI) were bias‐corrected percentile bootstrap intervals based on 1000 resamples of the 19 participants in the CGM group.

Receiver operating characteristics (ROC) analyses were performed to compare the accuracy of GA and HbA1c to detect poor glycaemic control defined as TIR <70%, TAB >25%, TBR >4% and TBR2 >1%. The area under the ROC curve (AUC) for each glycaemic marker was calculated as the Harrell's C statistic and presented with 95% CI adjusted for clustering. Optimal cut‐offs were estimated based on the Youden Index, and corresponding sensitivities and specificities were estimated in logistic regression models with random intercepts to allow for clustering. The statistical analyses were performed using IBM SPSS Statistics for Windows, version 26 (IBM Corp.) and Stata/SE for Windows, version 17.0 (StataCorp LLC).

## 3 RESULTS

In all, 42 women were asked to participate in the study and 41 were included. One participant withdrew during the study period, resulting in a total study population of 40 pregnant women. Among these, 26 (65%), 13 (32.5%) and one (2.5%) had type 1 diabetes, type 2 diabetes and maturity onset diabetes of the young (MODY), respectively.

In total, 17 women were CGM‐users before pregnancy. Out of the four women offered CGM during pregnancy, one delivered prematurely a week later. For another woman, the CGM raw data were lost, resulting in 19 women with available CGM‐data from first trimester to pregnancy week 36. The majority in the CGM group had T1D, whereas the non‐CGM group was more heterogeneous. All insulin‐pump users were in the CGM group, and most had Animas vibe pumps (Animas Corporation), while three women had either a Paradigm 715 (Medtronic), Minimed 640G (Medtronic) or an Omnipod (Insulet) pump. In contrast, most women used insulin pens in the non‐CGM group. Moreover, women in the CGM group were younger and had longer diabetes duration compared with the non‐CGM group. Pre‐pregnancy HbA1c level, BMI and weight‐gain in pregnancy were comparable between the two groups.

Almost one in five women developed preeclampsia, one third delivered an LGA‐newborn and two thirds had a vaginal delivery. The clinical characteristics of the total study population, CGM group and non‐CGM group are summarized in Table 1.

**TABLE 1:** Maternal and neonatal characteristics in the total study population, CGM‐group and non‐CGM group.

	Total study population (*n* = 40)	CGM group (*n* = 20)	Non‐CGM group (*n* = 20)	*p‐*value
Age, years	30.9 ± 5.5	29.2 ± 5.0	32.6 ± 5.5	.049*
Pre‐pregnancy BMI, kg/m^2^	25.8 (8.0)	25.8 (6.3)	25.8 (11.3)	.99
Pre‐pregnancy HbA1c, %	6.9 (1.3)	7.0 (1.3)	6.6 (1.3)	.99
Pre‐pregnancy HbA1c, mmol/mol	51.5 (15)	55.5 (15)	49.0 (15)	.78
Weight‐gain in pregnancy, kg	14.3 (8.9)	14.3 (8)	14.5 (9.7)	.78
Diabetes duration, years	10.5 ± 7.4	15.3 ± 6.5	5.0 (6)	<.001**
Nulliparous	35	40	30	.51
Retinopathy	33	50	15	.018*
Nephropathy	‐	‐	‐	‐
Chronic hypertension	5	10	‐	.15
Gestational age at inclusion (weeks)	12.4 ± 0.9	12.3 ± 0.7	12.6 ± 1.1	.27
Ethnic background				
European	78	90	65	.058
Middle Eastern	5	‐	10	.15
Asian	10	10	10	1.00
African	8	‐	15	.072
Diabetes type				
Type 1 diabetes	65	95	35	<.001**
Type 2 diabetes	33	5	60	<.001**
MODY diabetes	3	‐	5	.31
Anti‐glycaemic therapy in pregnancy				
Insulin	90	90	90	1.00
Metformin	5	5	5	1.00
Insulin and Metformin	5	5	5	1.00
Insulin pump	30	60	‐	<.001**
Pregnancy outcome				
Gestational age, weeks	38.9 (1.9)	38.9 (1.3)	38.9 (2.4)	.84
Preeclampsia	18	25	10	.21
Gestational hypertension	3	5	‐	.31
Preterm delivery	15	10	20	.38
Induction of labour	70	55	85	.038*
Vaginal delivery	60	40	80	.010*
Shoulder dystocia	‐	‐	‐	‐
Elective caesarean section	3	5	‐	.31
Acute caesarean section	38	55	20	.022*
Neonatal characteristics				
Birthweight, g	3794 (697)	3865 (726)	3683 (862)	.13
Birthweight, percentile	83.9 (42.2)	88.2 (28.7)	70.9 (42.8)	.040*
Large for gestational age	33	40	25	.31
Small for gestational age	5	‐	10	.15
NICU admission	43	50	35	.34

*Note*: Continuous variables are reported as mean ± SD or median (IQR) as appropriate, categorical data as percent.

Abbreviations: BMI, body mass index; HbA1c, glycated haemoglobin A1c; MODY, maturity‐onset diabetes of the young; NICU, neonatal intensive care unit.

*p < .05, **p < .001.

The majority (82.5%) completed all six blood samples for analyses of GA and HbA1c, whereas five women (12.5%) missed one blood sample and two women (5%) missed two blood samples. The main reason for not completing all blood samples was premature delivery. In total, 231 blood samples across gestation were available for analyses of GA and HbA1c.

After exclusion of six 14‐days periods with <70% coverage, 103 14‐days periods throughout gestation were available for the analysis of CGM‐data (mean coverage 92.6%, SD 4.9). The CGM metrics and laboratory markers of glycaemia varied across gestation (Table 2). We found correlations between GA and mean glucose, TIR, TAR and glucose SD (Table 3). For HbA1c, correlations were found with mean glucose, TAR, TBR and TBR2 (Table 3).

**TABLE 2:** Glycated albumin, HbA1c and CGM metrics across gestation.

	12 weeks	20 weeks	24 weeks	28 weeks	32 weeks	36 weeks
CGM metrics						
Mean glucose, mg/dl	119 (112, 128)	119 (110, 126)	119 (112, 128)	121 (114, 130)	121 (112, 130)	117 (108, 128)
Mean glucose, mmol/L	6.6 (6.2, 7.1)	6.6 (6.1, 7.0)	6.6 (6.2, 7.1)	6.7 (6.3, 7.2)	6.7 (6.2, 7.2)	6.5 (6.0, 7.0)
TIR, %	59 (54, 65)	63 (57, 68)	61 (55, 66)	61 (55, 66)	64 (58, 69)	68 (62, 74)
TAR, %	29 (23, 35)	27 (21, 33)	29 (23, 35)	31 (24, 37)	29 (22, 35)	25 (18, 32)
TBR, %	12 (8, 15)	10 (7, 13)	10 (7, 14)	9 (5, 12)	8 (4, 11)	7.2 (4, 11)
TBR2, %	7 (4, 10)	5 (3, 7)	6 (3, 9)	5 (3, 7)	4 (1, 7)	4 (2, 6)
Coefficient of variation, %	40 (37, 43)	38 (35, 40)	37 (34, 39)	36 (33, 38)	35 (32, 38)	34 (32, 37)
Glucose SD, mmol/L	2.7 (2.4, 2.9)	2.5 (2.2, 2.7)	2.5 (2.2, 2.7)	2.4 (2.2, 2.6)	2.3 (2.1, 2.6)	2.2 (2.0, 2.5)
Laboratory glycaemic markers						
CGM‐group (*n* = 20)						
Glycated albumin, %	12.1 (11.3, 13.0)	12.4 (11.5, 13.3)	11.3 (10.4, 12.2)	11.0 (10.1, 11.9)	10.2 (9.3, 11.1)	9.3 (8.4, 10.3)
HbA1c, %	6.1 (5.8, 6.4)	5.8 (5.6, 6.1)	5.7 (5.4, 6.0)	6.2 (5.6, 6.3)	6.1 (5.8, 6.4)	6.1 (5.8, 6.5)
HbA1c, mmol/mol	44 (40, 47)	40 (37, 44)	39 (36, 42)	44 (38, 45)	43 (40, 47)	44 (40, 47)
Non‐CGM group (*n* = 20)						
Glycated albumin, %	11.6 (10.2, 12.9)	11.1 (9.7, 12.5)	10.3 (8.9, 11.8)	10.0 (8.6, 11.5)	10.0 (8.5, 11.5)	9.2 (7.6, 10.8)
HbA1c, %	6.4 (6.0, 6.9)	6.0 (5.5, 6.4)	5.8 (5.4, 6.3)	5.9 (5.4, 6.4)	6.1 (5.5, 6.7)	6.1 (5.6, 6.7)
HbA1c, mmol/mol	47 (42, 52)	42 (37, 47)	40 (35, 45)	41 (35, 46)	43 (37, 49)	44 (37, 50)

*Note*: Data presented as mean with 95% confidence intervals, adjusted predictions. CGM metrics were calculated from 103 14‐days periods across gestation with >70% coverage.

Abbreviations: CGM, continuous glucose monitoring; HbA1c, glycated haemoglobin A1c; SD, standard deviation; TAR, time above range >140 mg/dl (>7.8 mmol/L); TBR, time below range <63 mg/dl (<3.5 mmol/L); TBR2, time below range <54 mg/dl (<3.0 mmol/L); TIR, time in range 63–140 mg/dl (3.5–7.8 mmol/L).

**TABLE 3:** Correlation coefficients with 95% confidence intervals for laboratory glycaemic markers and CGM metrics across gestation in diabetic pregnancies.

	Glycated albumin	HbA1c
Time in range (TIR)	**−0.58 (−0.77, −0.27)**	−0.41 (−0.66, 0.09)
Time above range (TAR)	**0.56 (0.35, 0.71)**	**0.58 (0.22, 0.77)**
Time below range (TBR)	−0.09 (−0.47, 0.25)	**−0.44 (−0.64, −0.14)**
Time below range 2 (TBR2)	−0.05 (−0.41, 0.26)	**−0.38 (−0.58, −0.11)**
Mean glucose	**0.49 (0.28, 0.62)**	**0.63 (0.32, 0.79)**
Standard deviation (SD)	**0.58 (0.24, 0.77)**	0.38 (−0.14, 0.66)
Coefficient of variation (CV)	0.36 (−0.09, 0.65)	−0.07 (−0.43, 0.22)

*Note*: Correlation coefficients for repeated measures design with 95% confidence intervals. CGM metrics were calculated from 103 14‐days periods across gestation with >70% coverage. Significant correlations are marked in bold.

Abbreviations: CGM, continuous glucose monitoring; HbA1c, glycated haemoglobin A1c.

**FIGURE 1:**
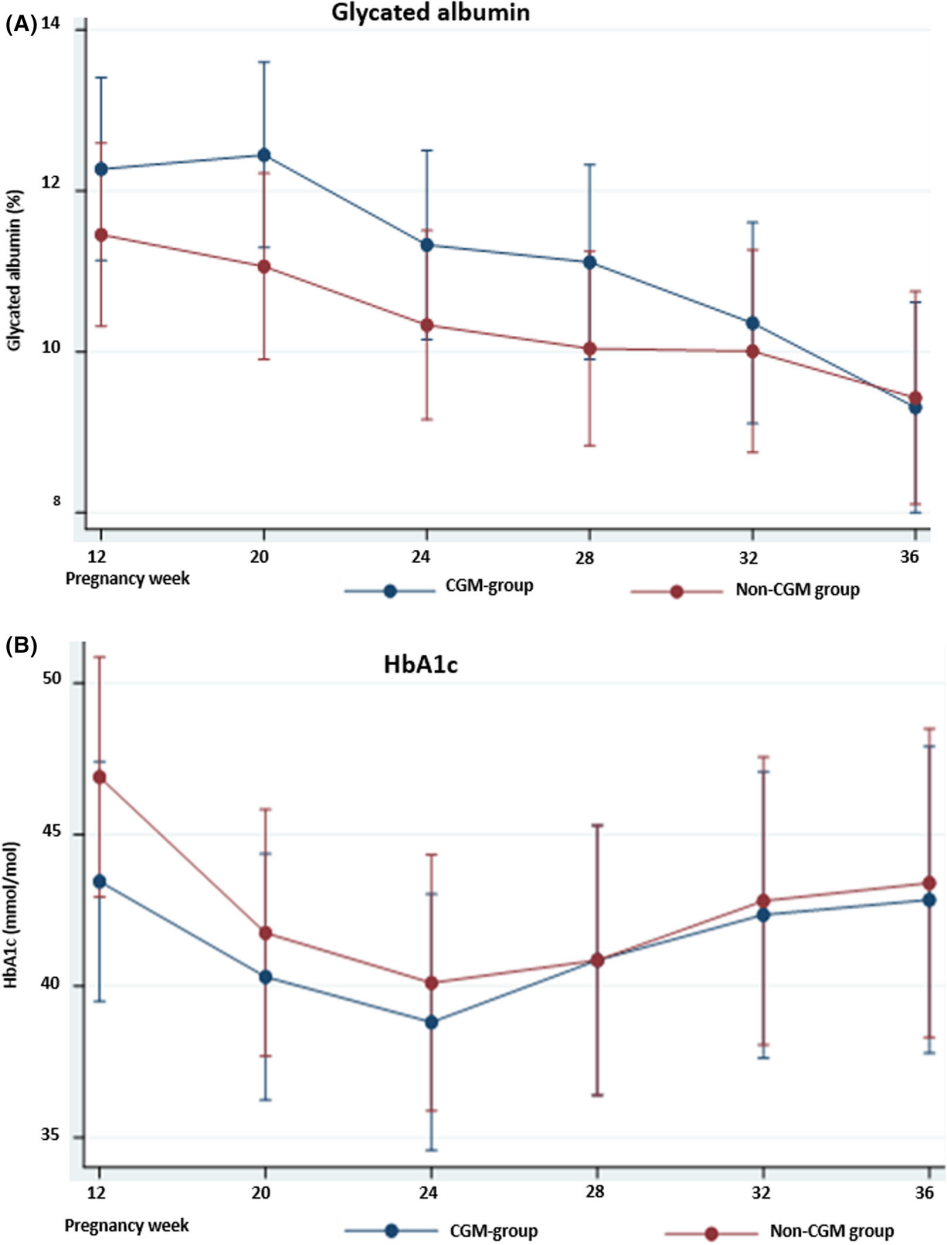
Glycaemic markers across gestation in the CGM and non‐CGM group. (A) Glycated albumin (%). (B) HbA1c (mmol/mol). Data presented as mean with 95% confidence intervals. CGM, continuous glucose monitoring; HbA1c, glycated haemoglobin A1c

The mean GA level decreased throughout gestation in both the CGM and non‐CGM group (Figure 1A), whereas the mean HbA1c level decreased from first trimester until pregnancy week 24, and increased towards pregnancy week 36 (Figure 1B), all changes statistically significant (*p* < .05).

**FIGURE 2:**
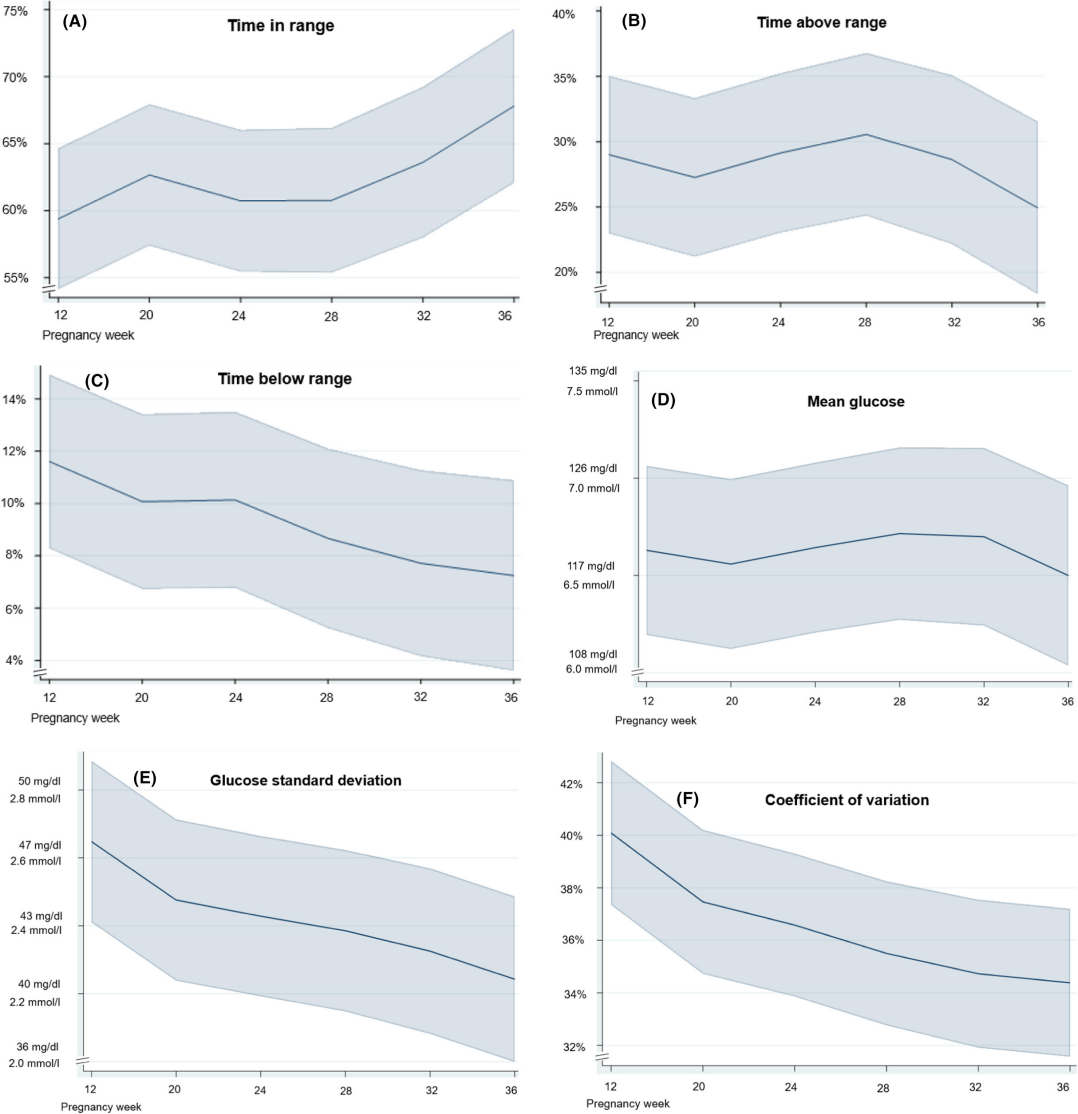
CGM‐metrics across gestation. (A) Time in range: 63–140 mg/dl (3.5–7.8 mmol/L). (B) Time above range: >140 mg/dl (>7.8 mmol/L). (C) Time below range: <63 mg/dl (<3.5 mmol/L). (D) Mean glucose. (E) Glucose standard deviation. (F) Coefficient of variation. Calculations based on 103 14‐days periods with >70% coverage. Data presented as mean with 95% confidence intervals, adjusted predictions. CGM, continuous glucose monitoring

Glycaemic control improved across gestation with more time spent in target range (Figure 2A) and less time spent above range and below range areas (Figure 2B,C). Mean glucose varied slightly (Figure 2D), whereas glycaemic variability decreased markedly (Figure 2E,F). However, in total, only 25 of the 14‐days periods (24%) achieved the international recommendation of >70% TIR for the pregnancy glucose target 63–140 mg/dl (3.5–7.8 mmol/L). For TAR <25%, TBR <4% and TBR2 <1%, the corresponding percentages were 38%, 28% and 19%, respectively.

**FIGURE 3:**
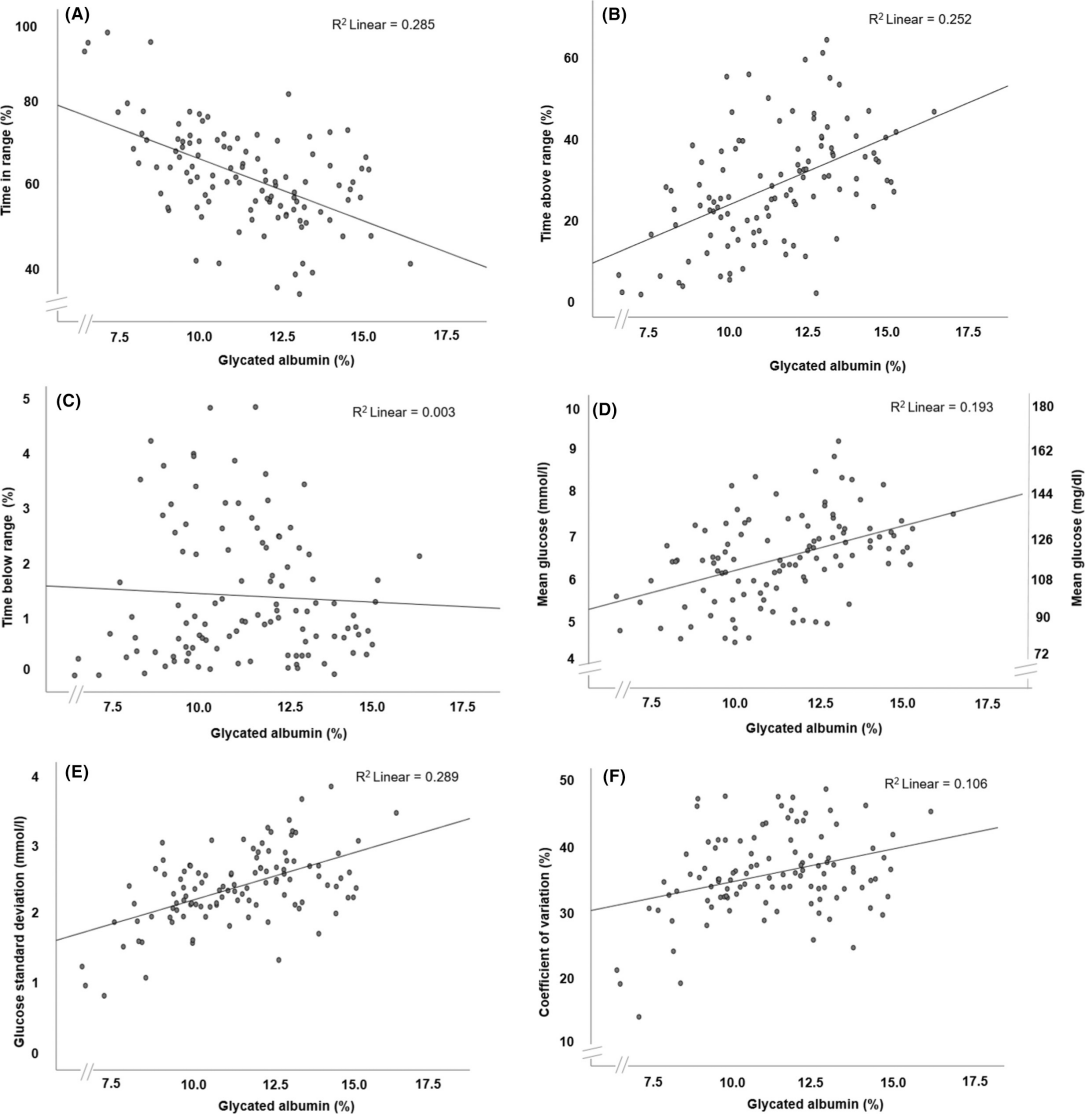
Scatterplots indicating the association between glycated albumin with CGM metrics. (A) Time in range: 63–140 mg/dl (3.5–7.8 mmol/L). (B) Time above range: >140 mg/dl (>7.8 mmol/L). (C) Time below range: <63 mg/dl (<3.5 mmol/L). (D) Mean glucose. (E) Glucose standard deviation. (F) Coefficient of variation. CGM metrics are calculated from 103 14‐days periods with >70% coverage. CGM, continuous glucose monitoring; *R* ^2^, coefficient of determination

We observed positive associations between GA and TAR, mean glucose, SD and CV (Figure 3B,D–F), a negative association with TIR (Figure 3A) and no association with TBR (Figure 3C). Corresponding scatterplots showing the association between HbA1c and CGM‐metrics are presented in Figure S1.

Receiver operating characteristic curves were used to assess the accuracy of GA and HbA1c to detect poor glycaemic control defined as non‐achievement of the clinical targets for CGM metrics, thus, TIR <70%, TAB >25%, TBR >4% and TBR2 >1% for the pregnancy glucose target 63–140 mg/dl. The adjusted AUCs for GA in detecting TIR <70%, TAB >25%, TBR >4% and TBR2 >1% were 0.78 (95% CI 0.60–0.95), 0.82 (95% CI 0.70–0.94), 0.56 (95% CI 0.31–0.82) and 0.66 (95% CI 0.42–0.90), respectively.

**FIGURE 4:**
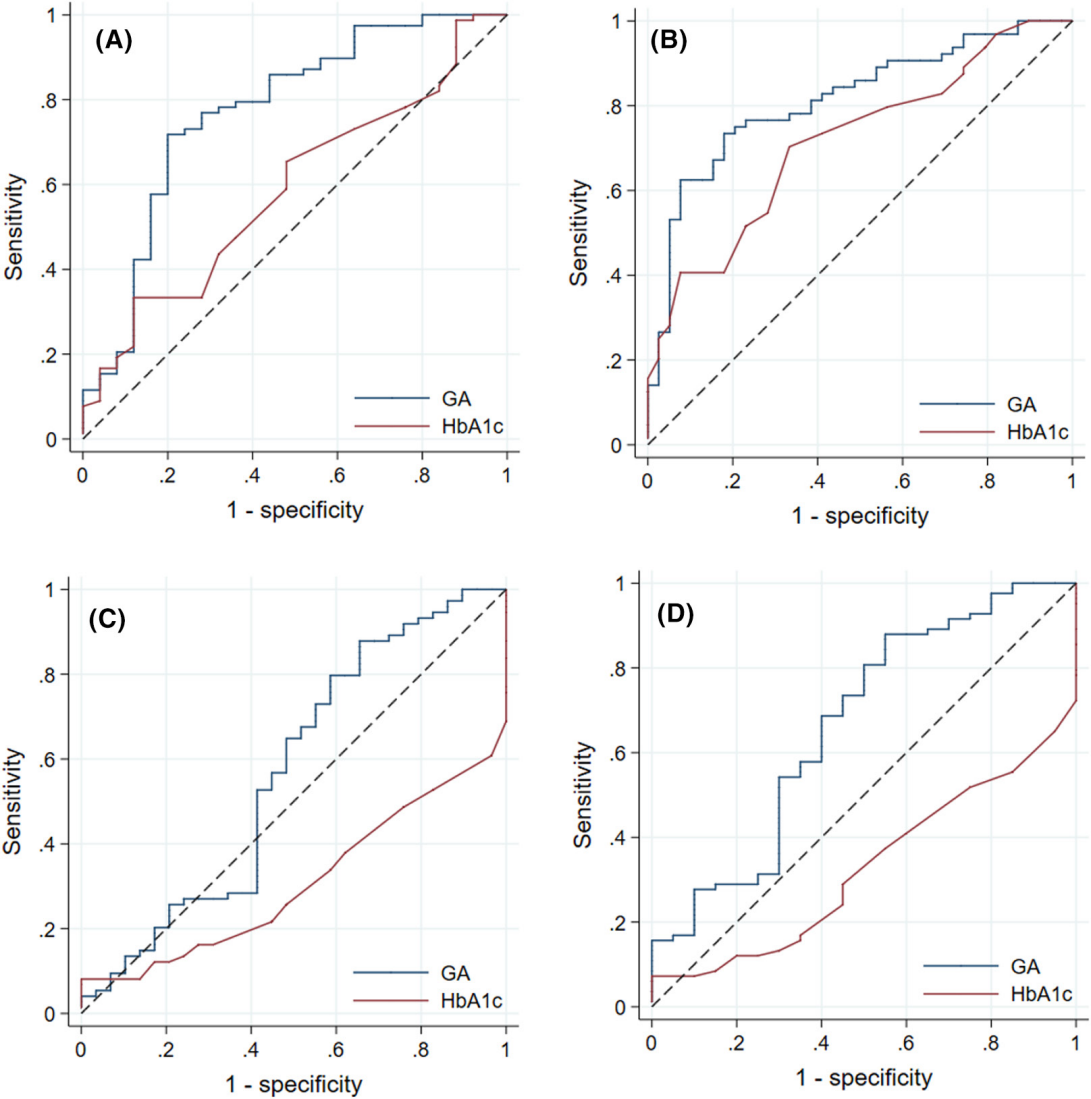
Receiver operating characteristic (ROC) curves to assess the ability of GA and HbA1c to detect poor glycaemic control. (A) Time in range <70%. (B) Time above range >25%. (C) Time below range >4%. (D) Time below range 2 >1%. Continuous glucose monitoring metrics are calculated from 103 14‐days periods with >70% coverage.

For HbA1c, the adjusted AUCs for detecting TIR <70%, TAB >25%, TBR >4% and TBR2 >1% were 0.60 (95% CI 0.41–0.78), 0.72 (95% CI 0.54–0.90), 0.30 (95% CI 0.13–0.47) and 0.32 (95% CI 0.13–0.52), respectively. The ROC‐curves are presented in Figure 4.

The optimal GA cut‐off value for detecting TIR <70% was >10.5%, with corresponding sensitivity (SE) 68% (95% CI 52%–83%) and specificity (SP) 73% (51%–95%). Similarly, the optimal cut‐off for detecting TAR >25% was a GA level >11% (SE 70 [54%–87%], SP 79 [62%–96%]).

## 4 DISCUSSION

In this prospective study of pregnant women with pre‐gestational diabetes, overall glycaemic control improved across gestation with more time spent in target range, whereas glycaemic variability decreased. Glycated albumin level decreased throughout pregnancy and correlated significantly with CGM metrics. In the ROC analysis, GA was markedly better than HbA1c to detect TIR <70% and TAB >25% with AUC values of 0.78 and 0.82.

Our findings support the use of GA as a biomarker of glycaemia in pregnant women with diabetes. As long as CGM is not available for all pregnant women, a short‐term biomarker to supplement self‐monitoring of blood glucose is useful. With the known limitations of HbA1c, this biomarker should not be used to assess glycaemia in pregnant women.^23^ The improving glycaemic control throughout pregnancy observed in our study using CGM‐metrics as the reference standard, was not at all reflected in lower HbA1c levels, in contrast, GA levels decreased throughout the pregnancy. We found high, statistically significant correlation between GA and glucose SD. Although not statistically significant, the positive correlation between GA and glucose CV and an AUC >0.5 for TBR >4% and TBR2 >1%, are in further support of previous findings indicating that high GA may also detect glycaemic variability,^10^ including hypoglycaemic fluctuations.

Others have shown that the GA level also decreases during gestation in women with healthy pregnancies.^24^, ^25^ The reasons remain unexplained, but might be due to increased turnover of albumin and/or increased selective loss of GA through glomerular filtration.^25^ Although the GA‐values are not directly comparable due to different methods for GA‐analysis, the observed decrease in mean GA level in our study is more prominent (from 12.1% to 9.3%). In comparison, the mean GA level in healthy pregnant women was 9.5% at pregnancy week 24–28 in our previous study,^19^ whereas a mean GA level of 11.3% and 10.3% was found in the CGM and non‐CGM group at pregnancy week 24 the present study.

Another population where HbA1c has limitation, haemodialysis patients with diabetes, Divani et al.^26^ found higher accuracy for GA than HbA1c to detect TIR <50%. None of the glycaemic markers were able to detect TBR. In the current study, for GA, the AUC of 0.66 for TBR2 >1% was not statistically significant, however suggesting that high GA levels may detect hypoglycaemic excursions. In contrast, HbA1c detected TBR and TBR2 above thresholds with AUCs of 0.30 and 0.32 (the latter not statistically significant), that is high HbA1c levels indicate reduced risk for these CGM metrics.

Albeit an increase in mean percentage of time spent in target range from 59% in first trimester to 68% in third trimester, most women in our study were far from achieving the recommended target >70% for TIR. Only 24% of the analysed 14‐days periods achieved TIR >70%, while 38% of the periods were within the target <25% for TAR. This is despite close follow‐up according to clinical guidelines during pregnancy. Moreover, the mean pre‐pregnancy HbA1c for the total study population was 51.5 mmol/mol, suggesting adequate glycaemic control.

In the CONCEPTT study, a multicentre randomized controlled trial on CGM use in pregnancy, time in target range reached 68% in the third trimester, similar to our study.^6^ In contrast, they reported markedly lower TBR (3% vs. 7%) and slightly higher TAR (27% vs. 25%) in third trimester. In a Swedish cohort study of 186 women with type 1 diabetes, corresponding proportions for TIR, TAR and TBR in the third trimester were 60%, 34% and 7%, respectively.^27^ In addition, the mean glucose level and glycaemic variability measures were higher in all trimesters. Taken together, these results indicate that it is challenging to obtain the targets for glycaemic control during pregnancy. Closed‐loop insulin therapy have shown promising results to improve glycaemic control but is not yet included in clinical guidelines.^28^

Strengths of the current study include the real‐life setting, the prospective design and the quantity of CGM data, continuously collected from first trimester until pregnancy week 36. In contrast, other studies report CGM data from notably shorter time periods of pregnancy, even as short as 3‐days.^29^ Moreover, repeated measurements of GA and HbA1c were performed and CGM metrics according to international consensus were reported.^8^ Among eligible women, all except one wanted to participate in the study and only one woman withdrew during the study period. Blood sampling and preparation of samples were performed by trained study nurses at the Clinical Trial Ward, and all samples were analysed at the same laboratory. Limitations include the limited sample size. Most CGM‐users in the present study had the Dexcom G4 device. Novel generations of CGM sensors such as Dexcom G6 may be more accurate.^30^ Moreover, three women had different CGM systems, possibly influencing the results. Due to the current absence of CGM‐criteria for women with T2D, we included the only CGM‐user with T2D in the analyses.

In this longitudinal study on pregnant women with pre‐gestational diabetes, GA level correlated well with CGM metrics. The improved glycaemic control observed was reflected in lower GA levels, but not in lower HbA1c levels. Higher GA levels were associated with less time spent in target range, more time spent in the above range area and increased glycaemic variability. Moreover, our results support previous findings that GA detects glycaemic variability better than HbA1c. Despite close follow‐up during pregnancy in line with clinical guidelines, most women in our study did not achieve the clinical targets for CGM metrics. In the ROC‐analysis, GA was more accurate than HbA1c to detect TIR <70% and TAR >25%. Thus, our findings support the use of GA to assess glycaemia in pregnant women with diabetes. Finally, our findings illustrate that GA and HbA1c have different qualities in the monitoring of glycaemic control. More studies, with larger sample sizes are required to better understand the role of GA in diabetic pregnancies, and for establishing optimal cut‐off values for detecting poor glycaemic control.

## AUTHOR CONTRIBUTIONS

**Johanne Holm Toft:** Conceptualization (equal); data curation (lead); formal analysis (equal); funding acquisition (lead); investigation (lead); methodology (equal); project administration (lead); resources (equal); software (equal); supervision (supporting); validation (equal); visualization (equal); writing – original draft (lead); writing – review and editing (lead). **Ingvild Dalen:** Conceptualization (equal); data curation (equal); formal analysis (lead); investigation (equal); methodology (lead); project administration (supporting); software (equal); supervision (equal); validation (equal); visualization (equal); writing – original draft (supporting); writing – review and editing (supporting). **Øyvind Skadberg:** Conceptualization (equal); investigation (equal); methodology (equal); resources (equal); validation (equal); writing – review and editing (equal). **Lasse Gunnar Gøransson:** Conceptualization (equal); formal analysis (equal); investigation (equal); project administration (equal); resources (equal); supervision (equal); writing – original draft (equal); writing – review and editing (equal). **Inger Økland:** Conceptualization (equal); data curation (equal); funding acquisition (equal); investigation (equal); project administration (equal); supervision (equal); validation (equal); writing – original draft (equal); writing – review and editing (equal). **Inger Hjørdis Bleskestad:** Conceptualization (equal); data curation (equal); formal analysis (equal); investigation (equal); project administration (equal); supervision (equal); validation (equal); writing – original draft (equal); writing – review and editing (equal).

## CONFLICT OF INTEREST

All authors declare no conflict of interest.

## Supporting information


Figure S1

## Data Availability

The data that support the findings of this study are available on request from the corresponding author. The data are not publicly available due to privacy or ethical restrictions.
