# Supplementary material for: Glycated albumin and continuous glucose monitoring metrics across pregnancy in women with pre‐gestational diabetes
Source: Endocrinol Diabetes Metab. 2022 Sep 19;5(6):e376. doi: 10.1002/edm2.376 (PMC9659665; doi:10.1002/edm2.376)
Supplement: Supplementary file 1 — Figure S1 [file EDM2-5-e376-s001.zip › edm2376-sup-0001-legend.docx]

**Figure S1** Scatterplots indicating the association between glycated haemoglobin A1c with CGM metrsics. a) Mean glucose. b) Time in range: 63–140 mg/dL (3.5–7.8 mmoL/L). c) Time above range: >140 mg/dL (>7.8 mmoL/L). d) Time below range: <63 mg/dL (<3.5 mmoL/L). e) Coefficient of variation. f) Glucose standard deviation. CGM metrics are calculated from 103 14-days periods with >70% coverage. R^2^: coefficient of determination; CGM: continuous glucose monitoring.
