# Supplementary figures and images for: Glycated albumin and continuous glucose monitoring metrics across pregnancy in women with pre‐gestational diabetes
Source: Endocrinol Diabetes Metab. 2022 Sep 19;5(6):e376. doi: 10.1002/edm2.376 (PMC9659665; doi:10.1002/edm2.376)

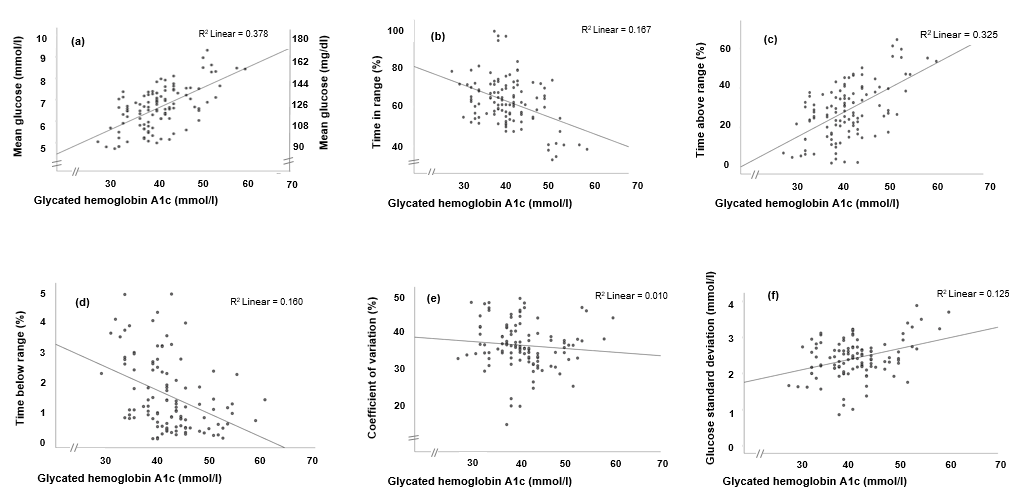

Supplement: Supplementary file 1 — Figure S1 [file EDM2-5-e376-s001.zip › edm2376-sup-0001-FigureS1.tiff]
